# Variola virus genome sequenced from an eighteenth-century museum specimen supports the recent origin of smallpox

**DOI:** 10.1098/rstb.2019.0572

**Published:** 2020-10-05

**Authors:** Giada Ferrari, Judith Neukamm, Helle T. Baalsrud, Abagail M. Breidenstein, Mark Ravinet, Carina Phillips, Frank Rühli, Abigail Bouwman, Verena J. Schuenemann

**Affiliations:** 1 Centre for Ecological and Evolutionary Synthesis (CEES), Department of Biosciences, University of Oslo, PO Box 1066 Blindern, 0316, Oslo, Norway; 2 Institute of Evolutionary Medicine, University of Zurich, Winterthurerstrasse 190, 8057 Zurich, Switzerland; 3 Institute for Bioinformatics and Medical Informatics, University of Tübingen, Sand 14, 72076 Tübingen, Germany; 4 School of Life Sciences, University of Nottingham, University Park, Nottingham NG7 2RD, UK; 5 The Royal College of Surgeons of England, 35-43 Lincoln's Inn Fields, London WC2A 3PE, UK

**Keywords:** virus evolution, smallpox, ancient DNA, metagenomics, museum specimens

## Abstract

Smallpox, caused by the variola virus (VARV), was a highly virulent disease with high mortality rates causing a major threat for global human health until its successful eradication in 1980. Despite previously published historic and modern VARV genomes, its past dissemination and diversity remain debated. To understand the evolutionary history of VARV with respect to historic and modern VARV genetic variation in Europe, we sequenced a VARV genome from a well-described eighteenth-century case from England (specimen P328). In our phylogenetic analysis, the new genome falls between the modern strains and another historic strain from Lithuania, supporting previous claims of larger diversity in early modern Europe compared to the twentieth century. Our analyses also resolve a previous controversy regarding the common ancestor between modern and historic strains by confirming a later date around the seventeenth century. Overall, our results point to the benefit of historic genomes for better resolution of past VARV diversity and highlight the value of such historic genomes from around the world to further understand the evolutionary history of smallpox as well as related diseases.

This article is part of the theme issue ‘Insights into health and disease from ancient biomolecules’.

## 1. Introduction

Smallpox was a highly contagious and lethal disease [1,2]. Before its eradication—declared in 1980 AD [1]—smallpox caused several large-scale epidemics that spanned centuries with remarkably high death rates [1,2]. For instance, between 1900 and 1980 AD, smallpox was responsible for an estimated 300–500 million deaths, or one in ten global deaths [3]. The causative agent of smallpox was the variola virus (VARV), a member of the genus *Orthopoxvirus* [1,4,5]. The variola virus is thought to have emerged fairly recently, around 3000–4000 years ago [6–9]. Historically, possible accounts of smallpox-like diseases have been recorded in 1122 BC China and 1500 BC India and rashes consistent with a smallpox infection have been observed in ancient Egyptian mummies dating to 1580–1100 BC [1,2,10]. The earliest unmistakable descriptions of smallpox, however, can first be found in the fourth century AD China, seventh century AD India and the Mediterranean, and tenth century AD southwestern Asia [1,2,11]. Despite the insights provided by historical accounts and molecular studies, the emergence of VARV and its subsequent evolution remain contested.

Ancient DNA (aDNA) studies can provide a new understanding of the emergence and evolution of diseases via the reconstruction of ancient pathogen genomes [12,13]. However, whereas several historical and ancient bacterial genomes are currently available, particularly for the causative agent of plague *Yersinia pestis* (e.g. [14–16]) or environmentally resistant mycobacteria (e.g. [17–21]), arguably less effort has been devoted to viral sequences. Early studies on historic viruses include, for example, the post-mortem detection of dolphin morbillivirus [22] and of the 1918 AD Spanish influenza virus [23,24]. Increased genomic resolution power later allowed for more in-depth phylogenetic analyses, e.g. of human immunodeficiency virus type 1 [25,26], and the reconstruction of complete RNA genomes, such as the 1918 AD pandemic influenza virus, [27,28] and barley stripe mosaic virus [29], as well as complete DNA genomes, such as hepatitis B virus [30–33].

Several attempts at obtaining VARV DNA sequences from historic tissues and materials have been undertaken [34] but only a few have been successful. Polymerase chain reaction (PCR) fragments isolated from seventeenth to eighteenth century AD Siberian mummies suggest a VARV origin approximately 2000 years ago [35]; however, the phylogenetic resolution that can be obtained with such short DNA sequences is limited. More recently, whole VARV genomes have been reconstructed from a seventeenth century AD Lithuanian child mummy [36] and from two specimens from the Czech National Museum in Prague dated to the nineteenth and early twentieth century AD [37]. Molecular clock analyses based on these genomes suggested somewhat contrasting dates for a common ancestor of all twentieth century AD circulating VARV strains, covering a time period between 1350 and 1645 AD [36–39]. Despite a lack of clear consensus on the exact dates, there is an agreement that the time of divergence between historic and twentieth century AD circulating strains predates the start of widespread vaccination in 1796 AD [1]. Furthermore, the divergence between P-I and P-II, the two clades which include all twentieth century AD VARV strains [11], also appears to predate modern vaccination, and the diversity within the modern clades seems to be as recent as the late nineteenth or early twentieth century AD. This suggests that VARV strains circulating at the time underwent a severe bottleneck as global smallpox vaccination programmes started at the turn of the twentieth century AD [1], resulting in the extinction of several older lineages [36].

During the eighteenth century AD, smallpox was endemic in Europe [3], with recorded increases in both frequencies of epidemics and mortality [40–42]. High mortality rates were recorded particularly for children, increasing from one in ten of all children burials in the second half of the seventeenth century AD to nearly one in three in the eighteenth century AD among London Quakers [43]. In order to capture more of the VARV diversity at this time and provide an additional calibration point for the dating of the common ancestor of modern and historic VARV strains, we computationally reconstructed an eighteenth century AD VARV genome from a museum specimen originally prepared by surgeon and anatomist John Hunter (1728–1793 AD) between 1760 and 1793 AD [44]. Phylogenetic analysis of our newly reconstructed historic VARV genome, together with all available historic and modern genomes, supports a recent common ancestor dated to the seventeenth century AD and suggests greater VARV diversity in the past than previously assumed. Our study also serves as an example of how increasing the number of available historic or ancient genomes can provide better resolution in phylogenetic inferences.

## 2. Material and methods

### (a) Sampling, DNA extraction, library preparation and sequencing

**Figure 1.**
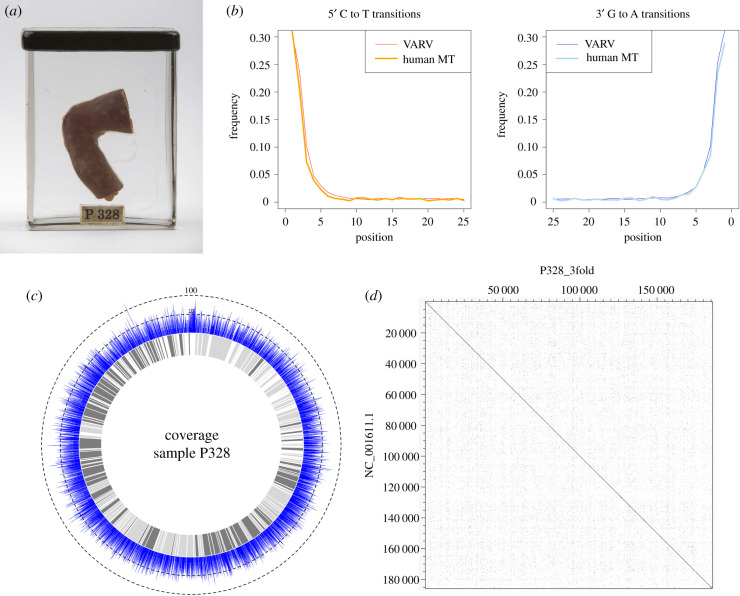
(*a*) Specimen RCSHC/P 328 showing smallpox lesions. Copyright © Museums at the Royal College of Surgeons. (*b*) Damage profile of reads mapping to the human mitochondrial genome (thick line) and the VARV reference genome (thin line). (*c*) Coverage diagram of the variola virus genome from sample P328. Blue indicates the coverage at a particular position (outer ring), coding areas of the genome are in grey (inner ring). The dotted circles indicate the coverage. (*d*) Conservation of genomic sequence between P328 and the variola virus reference genome NC_001611.1. The plot was generated with Dotter [45]. (Online version in colour.)

The sample used in this study was collected at the Hunterian Museum at the Royal College of Surgeons of England from specimen RCSHC/P 328, an ethanol-fixed infant leg embedded in liquid paraffin (figure 1*a*). The specimen is described as follows in the museum's online catalogue SurgiCat [44]: object name: ‘Leg, smallpox, Cases of Small Pox, Mounted wet tissue’; description: ‘Part of the leg from the same case as P327 showing smallpox lesions covering the surface of the limb. The epidermis has been reflected from the thigh to show the elevation of the lesions. This infant is likely to have contracted smallpox while *in utero*’. A thin cross section was sampled under clean conditions (i.e. tools and surfaces were cleaned with a sodium hypochlorite solution and protective equipment was worn to reduce contamination of the specimen by the workers). Only the cross section was transported to the University of Zurich. The tissue specimen was used in its entirety for DNA extraction and only DNA libraries are retained. The original specimen remained in the custody of the Royal College of Surgeons of England, from which it was not removed.

The DNA extraction was performed according to an adapted protocol by Devault and colleagues [46]. One hundred milligrams of tissue were homogenized using a sterile scalpel blade and incubated at 95°C and 1000 rpm for 5 min in 800 µl of extraction buffer (25 mM Tris-HCl, 5 mM CaCl_2_, 25 mM sodium citrate, 2.5 mM ethylenediaminetetraacetic acid, 1% sodium dodecyl sulfate, 50 mM 1,4-dithiothreitol, 10 mM N-phenacylthiazolium bromide). After adding 80 µl of proteinase K (20 mg ml^−1^), digestion was performed at 50°C and shaken for 24 h. Following centrifugation, the supernatant was extracted twice with a 25 : 24 : 1 phenol, chloroform and isoamyl alcohol mixture followed by a final chloroform step. DNA was isolated using QIAquick spin columns (QIAGEN), with two elutions in 30 µl elution buffer and reduced centrifugation speed (6–10 krpm) to prevent the loss of short DNA fragments. The DNA extraction was performed in an aDNA clean laboratory [47] following standard anti-contamination protocols [48–50] with parallel non-template control extractions.

Ten microliters of extract or extraction blank were used to generate Illumina sequencing libraries following a protocol optimized for aDNA [51,52] with modified P5 primers for compatibility with Illumina v4 sequencing chemistry. Two sample-specific indexes were added to each library via PCR amplification [52]. Blunt-end repair, adapter ligation and setup of indexing PCRs were performed in an aDNA clean laboratory. Six libraries were generated for sample P328 and non-template library blanks were generated in parallel. Indexed libraries went through 9–15 cycles of re-amplification in one or four 100 µl reactions containing one unit AccuPrime™ *Pfx* DNA polymerase (Thermo Fisher Scientific) or Herculase II Fusion DNA polymerase (Agilent), 1× AccuPrime™ *Pfx* reaction mix or Herculase II reaction buffer, 0.3–0.4 µM primers IS5 and IS6 [51] and 4–5 µl library template. Libraries were purified with MinElute spin columns (QIAGEN) following the manufacturer's instructions. Quantitative PCR [51] and analysis on an Agilent 2200 TapeStation were used to assess library quality and sequencing was performed on the HiSeq2500 and HiSeq4000 Illumina platforms with 2 × 125 + 7+ 7 or 2 × 75 + 8+ 8 cycles, respectively, by the Functional Genomics Center Zurich (Switzerland).

### (b) Risk assessment

All laboratory work was carried out under conditions stipulated by Swiss federal regulations (Swiss Federal Act on Research involving human beings. Human Research Act, HRA, RS 810.30). Owing to the age of the specimen and conditions of preservation, any identifiable DNA was expected to be damaged, highly fragmented and non-infectious [53,54] and no additional precautions were therefore taken. Furthermore, the DNA libraries were retained in a non-infectious form that cannot be used for reassembly owing to extended ligands on the ends of fragments of genetic material. In detail, the preparation process involved the addition of synthetic adapter molecules [51,52], typically much longer than the DNA fragments of VARV themselves, which would prevent any possible *in vitro* reassembly of the genome.

We followed the recommendations of the World Health Organization (WHO) for handling DNA from VARV samples [55] with the following exceptions: (i) owing to the highly fragmented and damaged nature of the aDNA [53,54], no autoclaving step of the sample and its byproducts was performed; (ii) temporary retention of greater than 20% of the VARV genome in a non-infectious and fragmented form with full extraction of sample and no remaining original material; and (iii) handling of the ancient VARV DNA in our dedicated laboratory at the University of Zurich. The WHO has viewed our handling procedure (as it had been carried out at the time the work was done) through a detailed risk assessment and supported this publication.

### (C) Data processing and analysis

#### (i) Metagenomic screening

The metagenomic screening of all six libraries of sample P328, as well as six non-template controls, was carried out with MALT v. 0.4.1 [56] using all complete bacterial, viral and archaeal genomes available in GenBank [57] as a reference (version May 2018). MALT was executed with the following mapping parameters: only reads with a minimum 85% identity (–minPercentIdentity) were considered as a possible match to the reference. Moreover, the minimum support parameter (–minSupport) was set to 5, i.e. only nodes with minimum support of five reads were kept. BlastN mode and SemiGlobal alignment were applied and a top per cent value (–topPercent) of 1 was set. All other parameters were set to default. For more details, see the electronic supplementary material, file S1. All contaminant genera previously detected are excluded from the resulting metagenomic composition [58]. MALT results were analysed and visualized using MEGAN6 v. 6.12.6 [59]. The ancient origin of the reads mapping to prevalent genomes was assessed by calculating damage profiles consisting of cytosine to thymine and guanine to adenine base misincorporation at the 5′ and 3′ ends of the fragments, respectively, typical for aDNA [53], using DamageProfiler v. 0.3.12 [60].

#### (ii) Read processing, mapping and variant calling

All sequenced libraries were processed using EAGER v. 1.92.55 [61]. To summarize, the sequencing quality was inspected with FastQC v. 0.11.5 [62], the reads were adapter trimmed and read-pairs merged into higher quality consensus sequences based on a minimum overlap of 10 bases with AdapterRemoval v. 2.2.1a [63] and subsequently aligned to the VARV reference genome (NC_001611.1) using BWA v. 0.7.17 [64] with a minimum quality score of 20 and a maximum edit distance of *n* = 0.01. Duplicates were removed with MarkDuplicates v. 2.15.0 [65], and DamageProfiler v. 0.3.12 [60] was used to investigate the damage patterns. The Genome Analysis Toolkit (GATK) v. 3.8.0 [66,67] was used to generate a mapping assembly and single nucleotide polymorphism (SNP) calling. The reference base was called if the position was covered at least three times and the quality score was at least 30. The base was called as an SNP if the quality score was at least 30 and 90% of the mapped reads contained this variant.

Furthermore, EAGER v. 1.92.55 [61] was also applied to map all reads against the human reference genome (GRCh37.p13) using the same parameters for quality assessment, adapter trimming and mapping as described above. Modern human DNA contamination was calculated using schmutzi [68] and the haplogroup was determined with HaploGrep2 v. 2.1.19 [69].

#### (iii) Phylogeny

A whole-genome alignment was calculated based on the newly sequenced and computationally reconstructed genome of sample P328, 44 modern and three historic publicly available VARV genomes [37,39,70–72], as well as eight additional *Orthopoxvirus* genomes (camelpox virus, taterapox virus, cowpox virus, horsepox virus, monkeypox virus, raccoonpox virus, skunkpox virus and volepox virus) [72–77] and a horsepox virus genome reconstructed from a vaccine manufactured in 1902 AD [78]. All accession IDs are listed in the electronic supplementary material, table S1. The sequences were aligned with MAFFT v. 7.407 [79] using the FFT-NS-2 algorithm. Based on the resulting alignment and using all sites, a maximum-likelihood tree was calculated using RAxML v. 8 [80] with 100 bootstraps.

#### (iv) Beast analysis

As there are discrepancies about the dating of samples V563 and V1588 [37,38], we used the Bayesian framework BEAST 2.5.2 [81] to estimate the tip dates for the historic genomes based on the time intervals given in the electronic supplementary material, table S2. These intervals cover the years from the original publication [37] and the corresponding comment [38]. We used uniform distributions for P328 (1760–1793 AD) and VD21 (1643–1665 AD) and normal distributions for V563 (mean: 1925, s.d.: 20) and V1588 (mean: 1929, s.d.: 60). Next, BEAST 2.5.2 was used to estimate divergence times and substitution rate based on 48 published VARV genomes [37,39,70–72], including strain P328. We excluded all non-human strains. The analysis was performed using a strict molecular clock, the K81 substitution model (bModelTest [82]) and assuming constant population size [83] as tested best previously [36]. For modern samples, the isolation dates were used as tip dates. The Markov chain Monte Carlo was run with 100 000 000 iterations rejecting the first 10 000 000 as burn-in. For more detailed parameters, see the electronic supplementary material, files S2 and S3.

## 3. Results

### (a) Sample information and processing

To computationally reconstruct the genome of an eighteenth century AD VARV strain, we collected a sample from a museum specimen with a known diagnosis of smallpox, specimen RCSHC/P 328 (later referred to as P328, figure 1*a*) from the Hunterian collection at the Royal College of Surgeons of England. The infant leg, whose surface is covered in smallpox lesions, was originally prepared by surgeon and anatomist John Hunter between 1760 and 1793 AD [44,84], and it is believed that the infant contracted the disease *in utero*. In 1780 AD, John Hunter published an account of a similar but unrelated case [85], where he describes the transmission of smallpox from a mother, who had recently recovered from smallpox, to her stillborn child. Following DNA extraction, we generated six P328 DNA libraries for shotgun sequencing, obtaining a total of approximately 150 million raw reads.

### (b) General metagenomic assessment

All P328 and non-template control libraries were processed the same way. First, all reads mapping to the human genome were excluded. The remaining reads were mapped against all available complete bacterial, viral and archaeal genomes in GenBank [57] using MALT [56] to monitor the metagenomic content in our dataset. The percentage of reads that could be assigned to the database per library varied from 0.49% to 31.11% (5.81% on average). The high number of unassigned reads may come from thus far unsequenced environmental bacteria. In general, P328 libraries are dominated by viruses (53.77%), followed by bacteria (45.47% on average) and archaea (0.76% on average) (electronic supplementary material, table S3). Moreover, we detected a high amount of reads mapping to Poxviridae (6.85%–24.38%), especially to the VARV genome (NC_001611.1) in all libraries except the non-template controls (electronic supplementary material, figure S1). To verify the ancient origin of the reads, the damage profiles of the reads mapping to VARV were determined (electronic supplementary material, figure S2). However, no specific metagenomic background could be assigned, which may be owing to the conservation of sample P328 in ethanol and paraffin. The top three species are plant-infecting viruses as well as bacteria specialized in the degradation of organic compounds such as *Methylotenera versatilis* without reliable damage profiles. Lastly, we also detected bacteria that were identified as contaminants in sequenced blanks [58].

We then merged all P328 libraries into a single file and separately mapped it against the VARV genome (NC_001611.1) and the human reference genome (GRCh37.p13) (electronic supplementary material, table S4). Based on the reads mapping to the human genome (62 250 656 reads), we were able to reconstruct 95.42% of the human mitochondrial genome with a mean coverage of 19.62 X and a damage profile of 28.07% to 30.68% (figure 1*b*), verifying the ancient origin of the sample. Next, contamination was calculated using schmutzi [68], resulting in 1% of modern-day human contamination. The haplogroup H3b1b1, which is common in Europe, was determined using HaploGrep2 v. 2.1.19 [69]. Furthermore, the analysis of reads mapping to the VARV reference resulted in a damage profile of 29.76% to 31.32% (figure 1*b*), which is consistent with the damage profile of the reads mapping to the human genome, further confirming the ancient origin of the reads.

### (c) Computational genome reconstruction and phylogeny

Using the merged shotgun sequencing data of all P328 libraries, 56 549 unique reads mapped to the VARV reference genome. Based on these reads, 85.18% of the VARV genome with a mean coverage of 14 X could be reconstructed with uniform coverage (figure 1*c*) using EAGER [61] (electronic supplementary material, table S4). Moreover, to investigate the synteny of strain P328, we compared it to the VARV reference genome, resulting in no major rearrangements and strong conservation of gene arrangements (figure 1*d*) [45].

**Figure 2.**
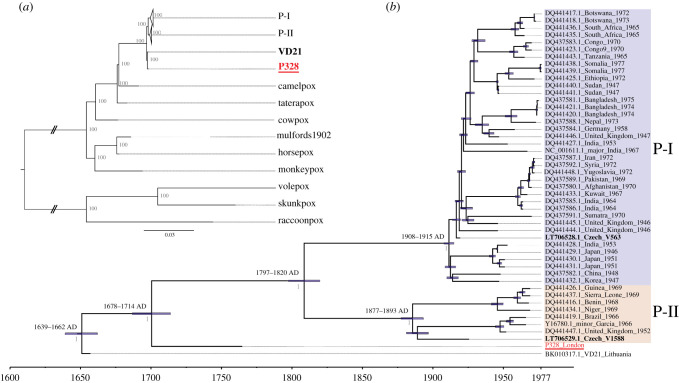
(*a*) Maximum-likelihood tree based on 57 *Orthopoxvirus* genomes. The historic genomes are bolded, the newly sequenced genome is in red and underlined. The bootstrap values are given as node labels in grey (100 BS). (b) Dated Bayesian maximum clade credibility tree reconstructed with BEAST 2.5.2 [81] (using a strict clock and constant population size). The nodes are labelled with the 95% HPD interval. Historic genomes are in bold, the newly added genome in red and underlined. Posterior values are given as node labels in grey. (Online version in colour.)

To access the phylogenetic placement of strain P328, a maximum-likelihood tree was calculated based on the whole-genome alignment of the newly sequenced genome of sample P328, 44 modern and three historic VARV genomes that were publicly available [37,39,70–72], as well as eight additional *Orthopoxvirus* genomes (camelpox virus, taterapox virus, cowpox virus, horsepox virus, monkeypox virus, raccoonpox virus, skunkpox virus and volepox virus) [72–77] and a horsepox virus genome reconstructed from a vaccine manufactured in 1902 AD [78] (figure 2*a*, the complete tree is shown in the electronic supplementary material, figure S3). The newly reconstructed genome falls, together with historic strain VD21 [36,39], basal to all modern VARV strains, which is consistent with previous studies [36,37,39]. Additionally, the root splits the pox strains into New and Old World *Orthopoxviruses* [74].

Owing to discrepancies in the dating of two historical samples from the Czech National Museum (V1588 and V563) [37,38], we used BEAST 2.5.2 to estimate the tip dates based on the intervals given by the two studies. We used a normal distribution around 1925 AD for V563 and around 1929 AD for V1588 as suggested by Porter *et al*. [38], but allowed the priors to cover the entire age range suggested by the two studies. These analyses resulted in the year 1925 AD for V1588 and 1920 AD for V563. The divergence times of the viral genomes were estimated based on the whole-genome alignment of 44 modern and three historic publicly available VARV genomes [37,39,70–72], together with the newly sequenced P328 genome using BEAST 2.5.2 [81] (figure 2*b* and table 1). We used a strict molecular clock with a constant population size and substitution model K81. The mean evolutionary rate of VARV is estimated to be 10.67 × 10^−6^ nucleotide substitutions per site per year. P328, dated to 1766 AD, forms a sister group to all modern human strains [70–72], as well as to the historic Lithuanian strain VD21 [36,39] dated to 1656. The time to the most recent common ancestor of VD21, P328 and all twentieth century AD VARV strains is estimated to 1651 AD (1639–1662, 95% highest posterior density (HPD)). The median divergence time of P328 and the modern strains is dated to 1701 AD (1687–1714 AD, 95% HPD). V563 and V1588 fall within the modern P-I and P-II clades, respectively, consistent with previous studies [37]. To test the robustness of the divergence time estimation, we reran the BEAST analysis excluding the strains V563 and V1588, resulting in almost identical divergence times (electronic supplementary material, figure S4).

**Table 1.** Comparison of the time to the most recent common ancestor (tMRCA) for the dated VARV phylogeny and individual branches for this and previously published studies [36,37,39]. (Dates are given in calendar years (AD). HPD, highest posterior density.)

branch splits, (AD)	this study		Duggan *et al*. [36]		Pajer *et al*. [37]	Smithson *et al*. [39]	
	mean tMRC	95% HPD	mean tMRCA	95% HPD	mean tMRCA	mean tMRCA	95% HPD
split VD21/P328/modern VARV	1651	1639–1662	1617	1588–1645	1350	1517	1470–1563
split P328/modern VARV	1701	1687–1714	na	na	na	na	na
split P-I/P-II	1809	1797–1820	1764	1734–1793	1695	1623	1579–1667
split P-I internal	1911	1908–1915	1910	1902–1917	1887	1881	1861–1897
split P-II internal	1886	1877–1893	1870	1855–1885	1808	1794	1754–1828

## 4. Discussion

Here, we successfully computationally reconstructed an eighteenth century AD VARV genome at a mean coverage of 14 X using shotgun metagenomic data. Because pathogen DNA typically represents only a very small fraction of an ancient or historic samples' metagenomic profile, hybridization capture is needed in most cases to reconstruct genomes (e.g. [14,36,46,86]). While this technique has the advantage of efficiently reconstructing pathogen genomes at a higher coverage and optimized sequencing costs, it can introduce bias, because evolutionary events such as genomic rearrangements, i.e. insertions, deletions, duplications, inversions and translocations, in addition to horizontal gene transfer, are likely to be missed when using extant genomes as a reference for hybridization probe design. Examples of genome reconstruction without enrichment for pathogen DNA exist [17,21,28,29,87–90] but represent a minority of aDNA studies. The P328 VARV genome we obtained without hybridization capture is highly consistent with the VD21 VARV genome obtained by Duggan *et al*. [36] using a capture approach, suggesting that this bias may be less of an issue, given the presence of strong genomic conservation such as for VARV [11,91]. This has also shown to be true for other ancient pathogen genomes, e.g. *Mycobacterium leprae* [17].

Besides investigating the species of interest, shotgun sequencing also provides the possibility to analyse the metagenomic composition of a sample by comparing it against a reference database. The top 10 identified species are dominated by plant-infecting viruses, such as the Dasheen mosaic virus, but these reads do not show a reliable damage profile. For the P328 libraries, the metagenomic analysis yielded a non-specific metagenomic background. This may be owing to a high content of unknown or unsequenced species, but also to the sample's conservation treatment in ethanol and paraffin and the lack of studies investigating the metagenomic content of this preservation method. Moreover, a relaxed identity parameter was applied when mapping to accommodate age-related DNA damage [53]. However, we clearly identified a high amount of reads mapping to the VARV genome, all showing a consistent damage profile, confirming the authenticity of the reads (electronic supplementary material, figure S2).

Phylogenetic analysis of the newly computationally reconstructed P328 VARV genome together with all available modern and historic VARV genomes [37,39,70–72], as well as eight modern and one historic *Orthopoxvirus* genomes [72–78], places P328 in a sister group to all twentieth century AD VARV strains together with the VD21 strain (figure 2*a*). This is consistent with the phylogenetic analysis reported by Duggan *et al*. [36]. Bayesian analysis assuming a strict molecular clock and constant population size suggests a common ancestor between 1639 and 1662 AD for all VARV strains included in this study [37,39,70–72]. This is consistent with the common ancestor suggested between modern strains and VD21, dated to between 1588 and 1645 AD [36], and clearly younger than the common ancestor dated to 1350 AD suggested by Pajer *et al*. [37].

Similarly, our phylogenetic analysis suggests a divergence between the P-I and P-II clades between 1797 and 1820 AD with P-I dating to between 1908 and 1915 AD and P-II to between 1877 and 1893 AD (figure 2*b*). While these dates are marginally younger than those reported by Duggan *et al*. [36], they are consistent with the hypothesis that the P-I and P-II clades diverged prior or contemporary to the development of smallpox vaccination in 1796 AD [1] (table 1) with evidence of a severe bottleneck following the rise in vaccination rates during the late nineteenth and early twentieth century AD. Furthermore, this is consistent with the observation that currently available twentieth century AD VARV genomes probably represent only a fraction of VARV genetic diversity in the past, because several strains are believed to have disappeared or were no longer detected owing to a loss in virulence [3]. Of the four historic strains included in our study, the younger V563 and V1588 [37] fall within the diversity of modern VARV strains, clustering in the P-I and P-II clades, respectively. By contrast, the seventeenth century VD21 [36,39] and the eighteenth century P328 both form their own sister groups to the twentieth century strains. This observation is consistent with modern VARV strains not being representative of past viral diversity and agrees with the scenario that selective pressure from increasing levels of vaccination caused several VARV lineages to disappear [3,36]. However, acquiring additional ancient and historic VARV genomes is crucial to capture the true diversity of VARV prior to the development of smallpox vaccination.

Finally, we addressed the discrepancies in dating of strains V563 and V1588, which were obtained from two specimens from the Czech National Museum in Prague [37]. These specimens were dated by Pajer *et al*. [37] to 60 and 160 years ago, respectively, but were contested to be of similar age and dating to the 1920s AD [38]. We used a tip calibration with normal distribution around the proposed younger dates, while allowing for the possibility of the specimens to be the age originally proposed. We obtained a mean date of 1920 AD for V563 and 1925 AD for V1588, consistent with the dates proposed by Porter *et al*. [38]. Furthermore, our proposed seventeenth century AD dating of the common ancestor between modern and historic strains is much closer to the sixteenth- to seventeenth century AD dating proposed by Duggan *et al*. [36] and Porter *et al*. [38] than to the fourteenth century AD dating obtained by Pajer *et al*. [37] when assuming an older date for strain V1588. Furthermore, excluding V563 and V1588 from the analysis still yielded similar dates (electronic supplementary material, figure S4).

An additional study by Smithson *et al*. [39] proposed an improved assembly for VD21, which when used in phylogenetic analysis, pushed back the date of the common ancestor between VD21 and modern VARV strains to the late fifteenth or early sixteenth century AD and the divergence between P-I and P-II to the late sixteenth or early seventeenth century AD (table 1). Here, we used the newly improved assembly for VD21 and obtained dates closer to those suggested by Duggan *et al*. [36] and older than those suggested by Smithson *et al*. [39].

The differing, and at times contested, dates assigned to VARV phylogeny presented here and in previous studies (e.g. [36,37,39]) show the importance of improving existing genomic assemblies, in addition to increasing the resolution power of such analyses by exploring and reconstructing additional historic genomes from an expanded spatio-temporal range. This is not only crucial to better understand pathogen evolution over a larger time transect and geographical distribution, but also to provide additional calibration points for phylogenetic analyses. To this end, metagenomic shotgun approaches, which do not use hybridization, such as the one presented here, can serve as a valuable tool to study a wide range of hosts and their associated viruses.

## Supplementary Material

Supplementary figures and tables

## Supplementary Material

Parameters used for MALT analysis

## Supplementary Material

BEAST configuration including samples from the Czech national museum

## Supplementary Material

BEAST configuration excluding samples from the Czech national museum

## Data accessibility

The raw sequencing data have been deposited at the European Nucleotide Archive (ENA) under project PRJEB35140 with accession IDs ERS3935826 - ERS3935837. The MALT parameters and the BEAST xml files used for our analyses have been uploaded as part of the electronic supplementary material.
