# Supplementary material for: Variola virus genome sequenced from an eighteenth-century museum specimen supports the recent origin of smallpox
Source: Philos Trans R Soc Lond B Biol Sci. 2020 Oct 5;375(1812):20190572. doi: 10.1098/rstb.2019.0572 (PMC7702794; doi:10.1098/rstb.2019.0572)
Supplement: Supplementary figures and tables [file rstb20190572supp1.pdf]

Supplementary Information

Variola virus genome sequenced from an eighteenth-century museum specimen supports the recent origin of smallpox

Giada Ferrari[1,2,†,*], Judith Neukamm[2,3,†,*], Helle T. Baalsrud[1], Abagail M. Breidenstein[2], Mark Ravinet[1,4], Carina Phillips[5], Frank Rühli[2], Abigail Bouwman[2,‡,*], Verena J. Schuenemann[2,‡,*]

1 Centre for Ecological and Evolutionary Synthesis (CEES), Department of Biosciences, University of Oslo, P.O. Box 1066 Blindern, 0316 Oslo, Norway.

2 Institute of Evolutionary Medicine, University of Zurich, Winterthurerstrasse 190, 8057 Zurich, Switzerland.

3 Institute for Bioinformatics and Medical Informatics, University of Tübingen, Sand 14, 72076 Tübingen, Germany.

4 School of Life Sciences, University of Nottingham, University Park, Nottingham NG7 2RD, UK.

5 The Royal College of Surgeons of England, 35-43 Lincoln's Inn Fields, London WC2A 3PE, United Kingdom.

† These authors contributed equally

‡ These authors jointly supervised this study

* *Correspondence: giada.ferrari@ibv.uio.no, judith.neukamm@uzh.ch, abigail.bouwman@uzh.ch, verena.schuenemann@iem.uzh.ch*

Supplementary Figures

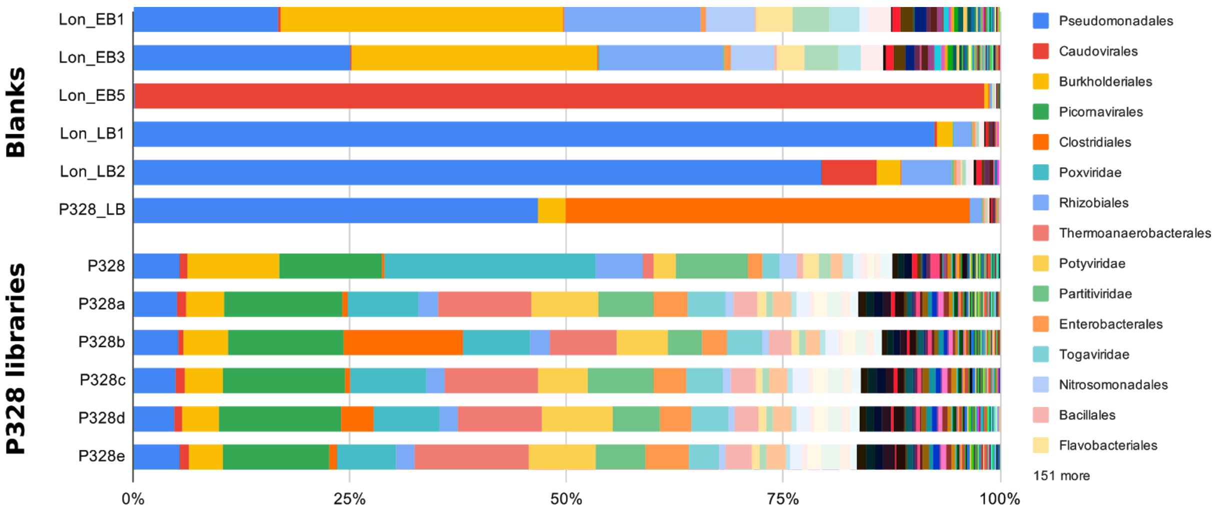

Supplementary Figure S1: Metagenomic composition of all libraries for sample P328, library and extraction blanks at the order level. All P328 libraries show a high amount of Poxviridae (turquoise), which is completely absent in the blank.

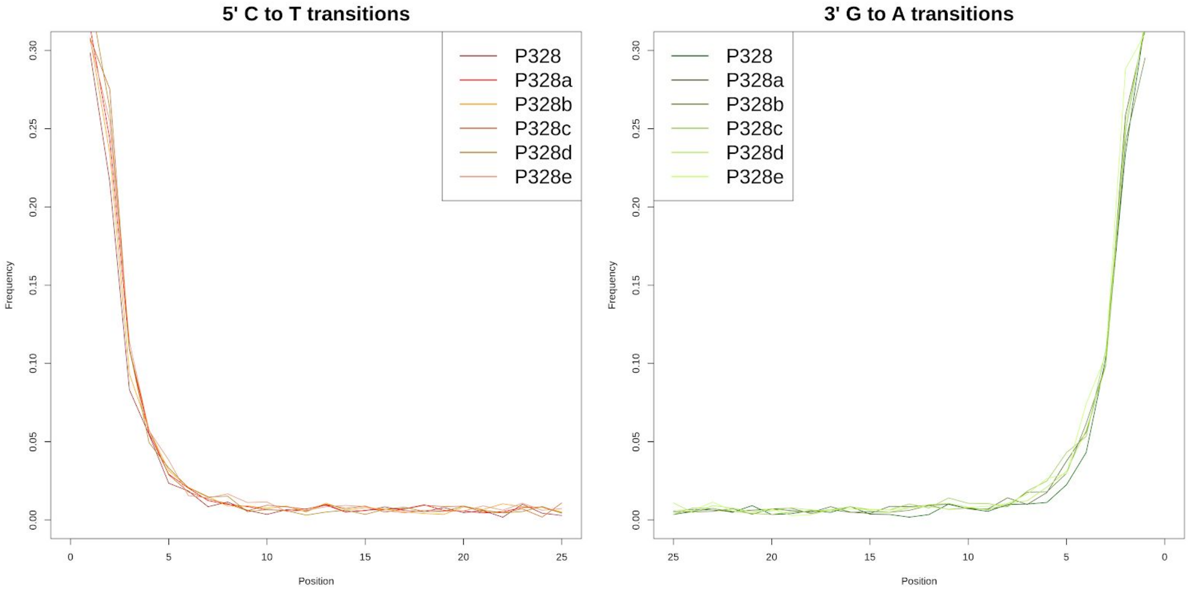

Supplementary Figure S2: Damage profiles of the reads mapping to VARV reference genome for all six P328 libraries. The reads were assigned with MALT to the VARV reference genome.

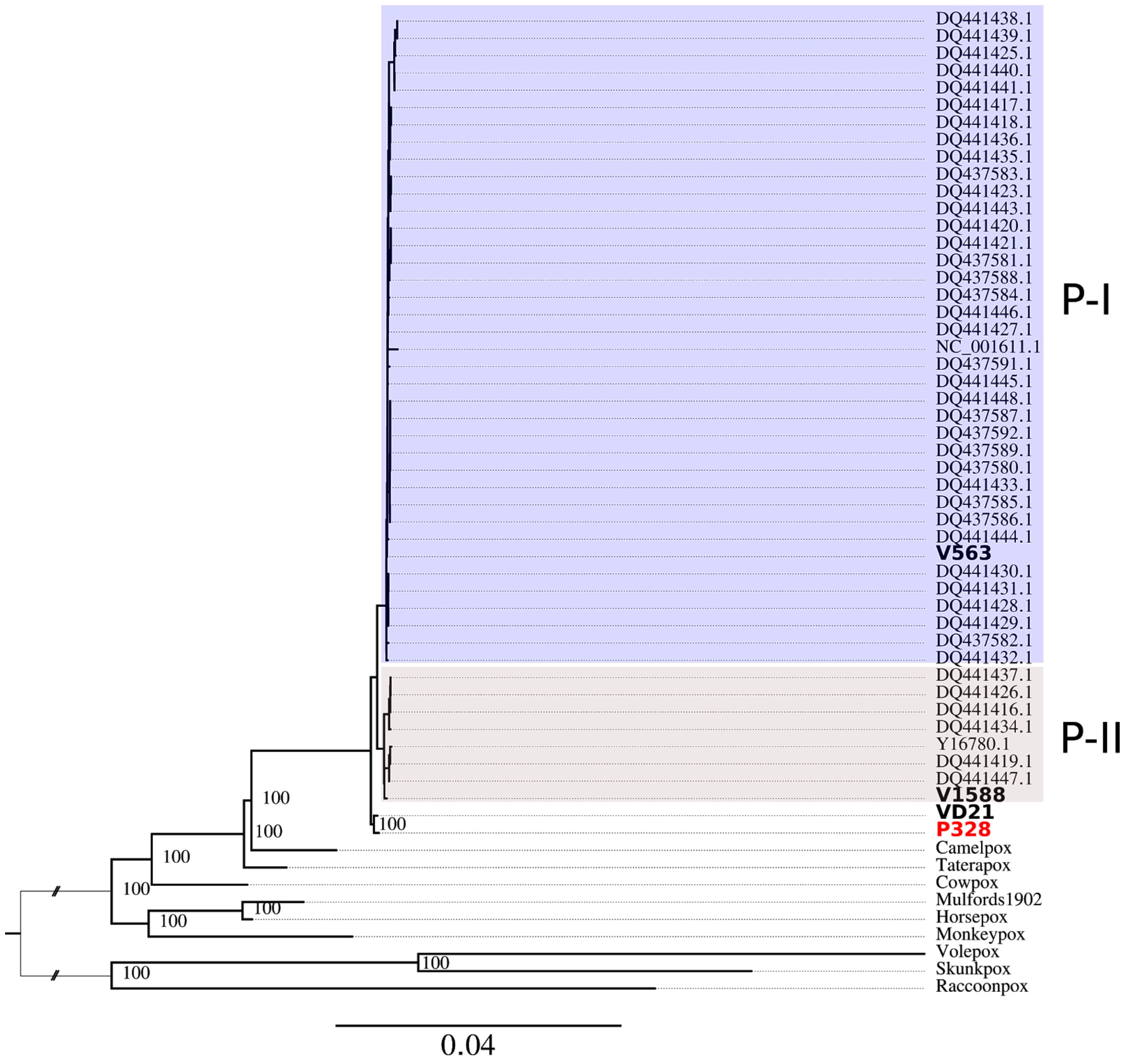

Supplementary Figure S3: Uncollapsed Maximum Likelihood tree including 57 Orthopoxvirus genomes. Bootstrap values are given as node labels. The historic genomes are in bold, the newly added genome in red.

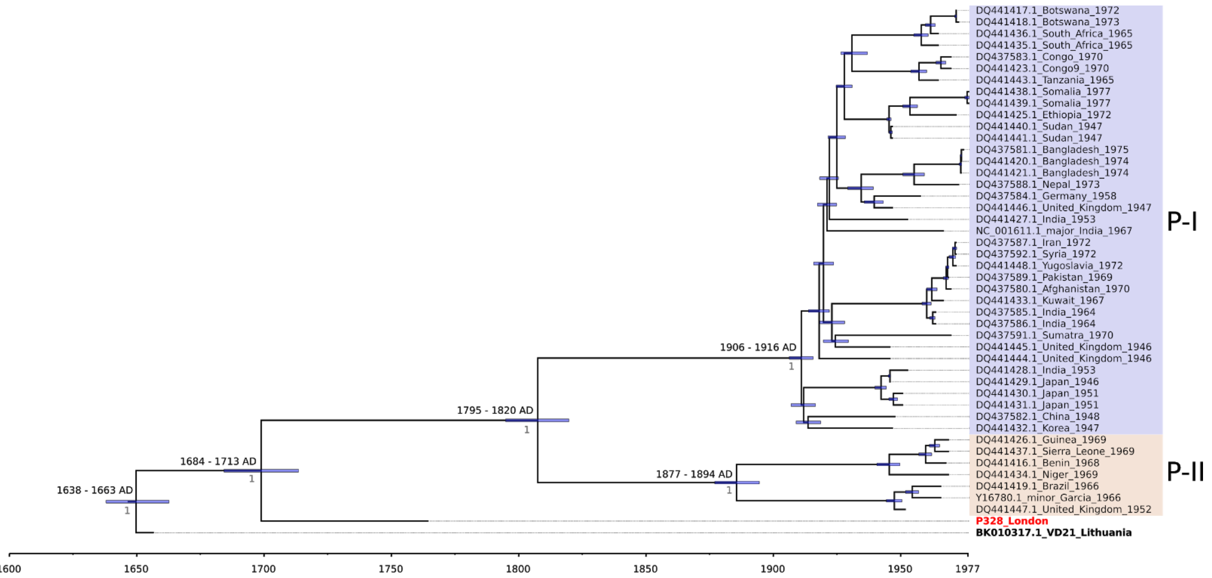

Supplementary Figure S4: Dated Bayesian Maximum Clade Credibility tree reconstructed with BEAST 2.5.5 (using a strict clock and constant population size) excluding strains V1588 and V563. The nodes are labelled with the 95% HPD interval. Historic genomes are in bold, the newly added genome in red. Posterior values are given as node labels in grey.
